# Does Big Mean Evil? Giant, but Benign Uterine Leiomyoma: Case Report and Review of the Literature

**DOI:** 10.1055/s-0040-1721351

**Published:** 2021-01-29

**Authors:** Luiz Gustavo Oliveira Brito, Natalia Lysei Ueno, Maira Rossmann Machado

**Affiliations:** 1 Department of Obstetrics and Gynecology, Faculdade de Ciências Médicas da Universidade de Campinas, Campinas, SP, Brazil; 2 Department of Gynecology and Obstetrics, Faculdade de Medicina de Ribeirão Preto da Universidade de São Paulo, Ribeirão Preto, SP, Brazil

**Keywords:** uterine leiomyoma, giant tumors, gynecological tumors, leiomioma, neoplasias ginecológicas, tumores ginecológicos

## Abstract

Uterine leiomyoma is the most prevalent benign type of gynecological tumor. It affects more than 80% of women worldwide and, within this group, more than 50% may be asymptomatic. However, large fibroid volumes may be associated with symptoms of extrinsic compression, and most of the cases do not present atypical cells. We present the case of a 49-year-old woman who underwent a total abdominal hysterectomy of a 13.5-kg uterine leiomyoma with no malignancies at histopathology and review the literature about giant uterine leiomyomas and their clinical repercussion. We concluded that large volumes do not always pose a threat regarding malignancy; however, future molecular studies are needed to investigate giant uterine fibroids.

## Resumo

Leiomioma uterino é o tumor benigno ginecológico de maior prevalência. Ele afeta mais de 80% das mulheres no mundo e, deste grupo, mais de 50% podem ser assintomáticas. Contudo, miomas de grande volume podem ser associados com sintomas de compressão extrínseca, e a maioria dos casos não apresentam células atípicas. Nós apresentamos o caso de uma mulher de 49 anos que foi submetida a histerectomia total abdominal de um espécime de 13,5 quilos sem malignidades ao exame histopatológico. Também revisamos a literatura sobre leiomiomas uterinos de grande volume e sua repercussão clínica. Concluímos que grandes volumes nem sempre representam um risco relacionado à malignidade; contudo, estudos moleculares futuros são necessários para investigar leiomiomas uterinos gigantes.

## Introduction

Uterine leiomyoma is a benign gynecological tumor originated from smooth muscle cells that affects up to 80% of women worldwide. From these, only 20 to 30% resent symptoms. 1 Multiple risk factors are associated with leiomyoma development, with the most common ones being nulliparity, obesity, early menarche, African descent, and age, all of them secondary to an extended exposure to estrogens or genetic predisposition. 1

Leiomyomas are classified as giant when their weight exceeds 11.4 kg. 2 They are included in the category of giant tumors, which is a concept that can also include ovarian tumors. Giant leiomyomas, in addition to the usually reported increase in abdominal size, presents other common clinical signs are mainly related to extrinsic compression. Examples of these signs and symptoms are worsening of the renal function, bowel obstruction, venous stasis, respiratory discomfort, thrombosis, and lymphedema. 1 Most of the cases only cause severe symptoms after a long period of growth. Herein, we describe the case of a patient with a growing abdominopelvic mass, the pre and postoperative results, and the immediate postoperative surgical evolution. Moreover, we present a review of the literature. The inclusion criteria for this review were all case reports on patients over 18 years old with uterine weight over 10 kg. We excluded all case reports with no data reporting weight and those whose authors did not answer about the weight of the specimen. The search strategy was composed of the following structure: (“uterine leiomyoma” OR “uterine fibroids” OR “uterine tumors”) (large OR giant), and the search was performed on January 3 ^rd^ , 2020 in the PubMed database with 577 results. Manuscript language was not considered a barrier for inclusion in this review. References were also consulted for analysis, as well as similar studies that were suggested in the PubMed website. Of these studies, 19 case reports that described giant leiomyomas were selected for analysis, and Table 1 displays detailed data for 14 of them. 2 3 4 5 6 7 8 9 10 11 12 13 14 15 16 17 18 19 20

**Table 1:** Giant leiomyomas (over 11.4 kg) – data obtained from the literature

Author	Uterine weight	Age (years)	Duration of symptoms	Symptoms	Preoperative assessment to rule out malignancy	Surgery and complications	Observations
Behrend 4	60.7 kg (133.8 lb)	35	9 years	Enlargement of the abdomen	Not performed	Removal of the tumor, no details	Patient died from pneumonia 48 hours after the surgery
Oelsner et al. 7	Two cases: 43.176 kg and 13.116 kg	49 and 54	10 and 7 years	1 ^st^ case - Slow progressive enlargement of the abdomen, respiratory insufficiency with intubation prior to surgery. 2 ^nd^ case - abdominal pain and enlargement, weight loss, cachexia	1 ^st^ case – urgent laparotomy and no pre-op performed 2 ^nd^ case – tumor size was big and patient could not undergo CT	1 ^st^ case – TAH-BSO 2 ^nd^ case – mass removal + TAH-BSO	1 ^st^ case evolved well. 2 ^nd^ case evolved with grand mal convulsions
Reddy et al. 8	17.69 kg (39 lb)	67	20 years	Increasing abdominal distension + low back pain. Tumor with air-fluid levels preop image	Only CT – no endometrial bx	TAH-BSO	Fibroid with septic degeneration – cefuroxime + metronidazole
Pérez and Ramón 9	27 kg	40	12 months	Weight loss, increasing abdominal distension	Normal tumor markers (did not specify). CT-guided fine-needle aspiration biopsy (mesenchymal tumor).	TAH-BSO + segment of jejunum	No malignancies – post op uneventful
Nappi et al. 10	27.7 kg		18 months	Abdominal enlargement, weight gain of 25 kg	MRI + US – multilocular mass, undetermined origin. Normal CEA, CA-125, AFP. No endometrial bx	TAH-BSO + omentectomy	None
Amber et al. 11	26.94 kg	47	3 years	Cachexia, giant abdominal mass	CT with contrast – suspicious of leiomyosarcoma. AFP, CA 19–9 and CEA with normal levels. CA-125 slightly elevated.	TAH-BSO, appendectomy and partial omentectomy	Reoperated after 24 hours – hemorrhagic shock – Discharge 3POD
Semczuk et al. 12	15.2 kg	50	Not reported	Bilateral hydronephrosis by abdominal tumor	No pre-op assessment.	TAH-BSO. Bilateral double-J catheters insertion before surgery	Coexisting with endometrial cancer deriving from a polyp
Savulescu et al. 13	18.1 kg	45	10 months	Increased abdominal size, constipation, urinary loss, urinary frequency, back pain	US and CT. No endo bx.	TAH-BSO	Discharge at POD #6;
Mate et al. 15	13.5 kg	71	Not informed	Increased abdominal distension, cachexia	CT. Normal CEA, AFP, B-HCG. CA-125 94 U/mL.	TAH-BSO	No complications
Orazulike et al. 16	15.5 kg	43	14 years	Increasing abdominal distention + pelvic pain	Only ultrasound. Normal CA-125. No endometrial biopsy	TAH-BSO	Discharged POD #7
Ezugwu et al. 17	16.8 kg	31	8 years	Progressive abdominal swelling, infertility, weight loss and dyspnea	Transabdominal US. Authors considered doing CA-125, CT and MRI but did not have funds to request for patient. No endo bx	Myomectomy	Elective cesarean section at 38 weeks
Moris and Vernadakis 18	28.1 kg	39	4 months	Progressive constipation, increasing abdominal size, back pain, urinary frequency	Transabdominal US and CT – no ascites, metastases, or enlarged pelvic or para-aortic lymph nodes. No endo bx	Midline incision – ASH-BSO – Postop uneventful – 10 ^th^ day	None
Sonoo et al. 19	17.4 kg	37	3 years	Fatigue for 2 weeks – Hyperkalemia (10.3) and arrived in CPA	Post-mortem laparotomy – not performed any pre-op	Post-mortem laparotomy for mass resection	Death
Lim et al. 20	27.8 kg	53	Not reported	Massive uterine mass complicating restrictive lung disease		TAH-BSO	Coagulopathy and hemorrhagic shock

**Abbreviations** : AFP, α-fetoprotein; ASH-BSO, supracervical abdominal hysterectomy + bilateral salpingo-oophorectomy; Bx, biopsy; CEA, carcinoembryonic antigen; MRI, magnetic resonance imaging; TAH-BSO, total abdominal hysterectomy + bilateral salpingo-oophorectomy; US, ultrasound.

## Case Report

**Fig. 1:**
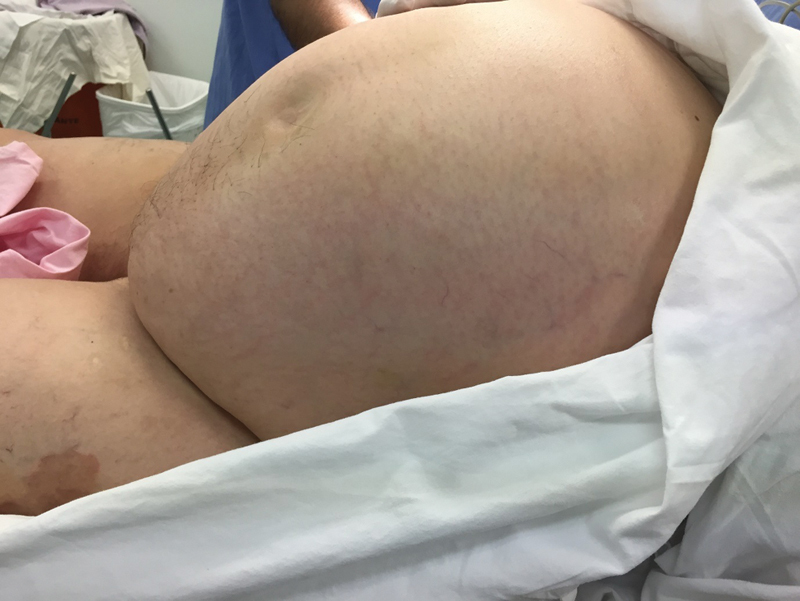
Giant mass occupying all abdomen.

A 49-year-old nulliparous, obese woman with chronic hypertension sought medical care due to an increase in the size of her abdomen ( Fig. 1 ) for one year. She was amenorrheic for about a year without the use of contraceptives, and, 6 months prior to her consultation, she had been submitted to an abdominal computerized tomography (CT), which showed a uterine mass measuring 25.7 × 29.0 × 29.5 cm (volume 11,432.9 cc). The patient presented with complaints of frequent urination and urinary incontinence, with a urinary infection diagnosed on screening laboratory evaluation. Physical examination revealed a palpable abdomino-pelvic mass located up to 2 cm below the xiphoid process. Gynecological examination was unaltered, and no malignancy was found in the pap smear.

**Fig. 2:**
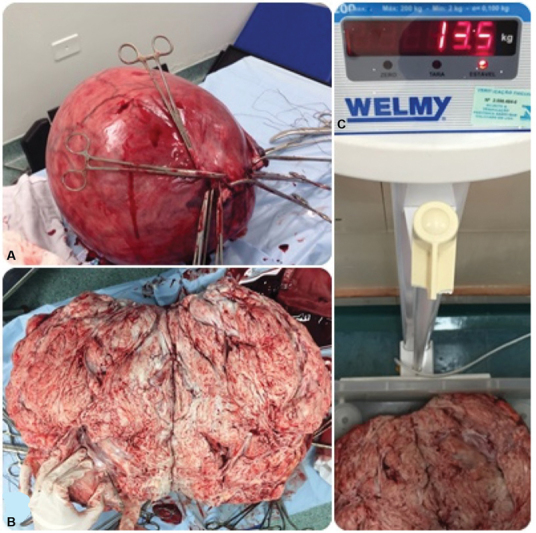
( **A** ) Voluminous uterus extracted after hysterectomy. ( **B** ) Macroscopic aspect of a leiomyoma (cut section). ( **C** ) Specimen weighing 13.5 kg.

As there were no previous radiological exams evaluating the uterine growth rate and we could not rule out leiomyosarcoma for this patient preoperatively (a preoperative endometrial sample was attempted – Pipelle de Cormier) without sufficient material, a total abdominal hysterectomy with bilateral salpingo-oophorectomy was scheduled. One week before surgery, the patient underwent a magnetic resonance imaging (MRI), and the size of the tumor did not present any alterations. No hypothesis for leiomyosarcoma was performed. One day before the surgery, the patient was complaining of progressive abdominal pain, constipation, and urinary retention. Low-molecular weight heparin (enoxaparin) was administered prophylactically prior to surgery. A median incision was performed, and surgery (total hysterectomy plus prophylactic salpingectomy) occurred with no abnormalities, with an estimated blood loss of 600 ml, measured by compress weighing. An enormous uterine mass, weighing 13.5 kg, was removed ( Fig. 2 ). Ureterolysis was performed to confirm that both ureters were intact.

Intraoperative histological analysis of the mass revealed no atypia, and the specimen was sent to pathology. No complications occurred in the immediate postoperative period, and the patient was discharged two days after the surgery. Gross macroscopy did not find any abnormalities, except for a giant uterine fibroid, with abundant vasculature in the periphery of the lesion. No areas of degeneration were seen. Histopathological examination evidenced leiomyoma with no atypia or degeneration and unaltered adnexa. The patient was discharged with no complications.

## Discussion

Giant leiomyomata are rare, and not frequently reported in the literature. They are of special interest when presenting with a fast growth rate, which suggests malignancy, as uterine cancers usually present with signs and symptoms similar to benign tumors of the uterus and there are no specific criteria for the suspicion of leiomyosarcoma. 1 The first report of these cases occurred in 1888, with the highest reported uterine tumor (63.6 kg), which was operated by Hunt et al. 3 after an autopsy. Behrend 4 operated a 60 kg tumor, and the patient died due to pneumonia 48 hours later. 4 Singhabhandhu et al. 5 have described the largest uterine tumor removed with survival of the patient (45.5 kg).

A case series ( *n* = 2) published by Oelsner et al. 7 has performed a literature review, and 57 reported cases were found up to 2003. After 2003, our review has identified 13 cases plus the one described in the present article, thus comprising 71 cases. Our table summarizes most of the recent cases, and, after 2003, the largest reported uterine leiomyoma weighed 28.1 kg. 18 The main complaint is the progressive enlargement of the abdomen, and, in some cases, serious malnutrition was observed, as well as complications related to other organs that were damaged due to the expanding abdominopelvic tumor. One of the reported cases 15 presented with a coexisting endometrial tumor, and only one case after the 2003 review evolved with death. 19

Surgery can be challenging in these cases. It is quite difficult to consider a minimally invasive approach in a large abdomen, as most of the incisions were xiphopubic. Within the selected cases, only one case was performed myomectomy—it was a nulliparous woman. 17 As the uterine tumor presents large dimensions, the standard treatment for uterine sarcomas (total hysterectomy + bilateral salpingo-oophorectomy) is suggested and performed. A multidisciplinary approach with other specialties (general surgeon, plastic surgeon, urology) may be needed to help the gynecological surgeon during this procedure.

The present case report does not show an association between tumor size and malignancy. We need to understand the biological behavior of significantly enlarged leiomyomas with molecular approach. A study comparing large leiomyomas versus simple uterine leiomyomas has shown that there are large growth rates are present in fibroids from adolescent women. 21 There is a systematic review studying uterine leiomyoma in adolescents, and the biological behavior of these tumors in women under 20 years seems to be different, because they present higher growth rates in adolescents than in adults. 22 In summary, there is a need for further elucidation on the relationship between giant uterine fibroids and their growth rate and the associated malignancy potential.
